# In India, most principal investigators have run very few trials over the years

**DOI:** 10.3389/fmed.2024.1424570

**Published:** 2024-08-07

**Authors:** Rishima Borah, Anwesha Dhal Samanta, Khujith Rajueni, Vina Vaswani, Gayatri Saberwal

**Affiliations:** ^1^Institute of Bioinformatics and Applied Biotechnology, Bengaluru, India; ^2^Research Unit of Population Health, Faculty of Medicine, University of Oulu, Oulu, Finland; ^3^Yenepoya (Deemed to be University), Mangaluru, India

**Keywords:** clinical trial registries, Clinical Trials Registry—India, interventional trials, trial transparency, trial ethics, trial governance, data quality, trial participant safety

## Abstract

**Background:**

In the past, clinical trials run in India have been the subject of criticism. Among other steps to improve the trial ecosystem, for some time the government limited the number of trials that a Principal Investigator (PI) could run to three at a time. We were interested to know how many trials PIs in India tend to run at a time.

**Methods:**

We accessed the 52,149 trial records hosted by the Clinical Trials Registry—India in April 2023. Of these, we shortlisted trials that had run in India, were interventional, and involved certain interventions such as drug, biological etc. We used multiple parameters, such as email ID, phone number etc. to determine whether one name always represented the same PI and whether two names corresponded to the same PI. We then determined how many trials each PI had run.

**Results:**

We found that 3,916 unique PI names were associated with 6,665 trials. Of these, 2,963 (75.7%) PIs had run a single study. Only 251 (6.4%) had run more than three trials. A mere 14 PIs had run 20 or more trials. The 14 PIs were affiliated with local pharma companies (6), local or global contract research organizations (4), multinational pharma companies (3) and the Central Council for Research in Homeopathy (1). The maximum number of trials run by a single PI was 108. Of these, the largest number run in a single year, 2022, was 53.

**Conclusion:**

Each PI name needs to be connected to a unique ID that does not change with time, so that it is easier to track the number of trials that a given PI has run. The number of studies run by a given PI at a given time must not be excessive and needs to be monitored more actively. The government needs to consider whether a cap on the number of trials that a PI runs at a time is required and what infrastructure needs to be in place to facilitate higher numbers of trials. Trial registry records need to be updated more regularly. Other countries may wish to do likewise.

## Introduction

The World Health Organization (WHO) recognizes 17 clinical trial registries as primary registries (1). These registries are hosted in countries around the world, from Australia in the east to Brazil and Peru in the west, and includes nations such as South Korea and Sri Lanka, and regions such as Africa and the European Union. The Clinical Trials Registry—India (CTRI) is one of the primary registries. Although the data in such registries has been put to many uses (2), one of their biggest functions is to provide transparency around ongoing trials. For instance, this information may alert the public to ongoing studies, such as those relating to COVID-19 (3, 4), that people may have wished to participate in during the COVID-19 pandemic. The information may also highlight problems with an ongoing study, such as one sponsored by a multinational company in India a few years ago that was found to break the law, leading to a governmental investigation (5). Additionally, since there were 70,126 trials registered with CTRI on 6 July 2024 (6), the availability of information related to a large and rapidly growing number of studies enables various types of analyses that can feed into policymaking.

In the past, clinical trials run in India have been the subject of some criticism. The accusations, from various non-governmental organizations in particular, culminated in a 2012 Parliamentary report (7), which was harshly critical of the office that regulates drugs and related clinical research in the country, the Central Drug Standards Control Organization, or CDSCO. A major criticism concerned the perceived too-close relationship between industry and the regulator. In response, in 2014 the government set up the Prof. Ranjit Roy Chaudhury Expert Committee to address several issues with respect to the regulation of drugs, including clinical trials. The committee’s report (8) made various recommendations to improve the clinical trial ecosystem. Although the report did not limit the number of trials that a given Principal Investigator (PI) could run at a time, the government limited this to no more than three (9). Henceforth, we refer to this as the “rule of three.”

In the context of this study and CTRI, the PI is the lead researcher named in the trial registry. The PI field that was used was “Details of Principal Investigator or overall Trial Coordinator (multi-center study).” It was not the site-specific PI data (where one or more PIs may be listed.). As such, he/she was presumed to bear responsibility for the overall design, conduct, and management of the clinical trial. However, the detailed responsibilities of this person are not specified in the descriptions of each field provided by CTRI (10).

In 2016, the rule of three was revoked (11, 12). The revocation of this rule appears to have been done after industry pushback, in order to improve “the ease of doing business” in India (13). It is now the Institutional Ethics Committees (IECs) that will decide whether or not a given PI may run a proposed trial, thereby giving the IECs immense power. This had been questioned at the time (13). Further, recently it has been reported that many of the ECs are dysfunctional (14) further strengthening the case that the ECs should not have this much power.

In 2011, it was noted with concern that there was a PI who had run as many as 25 trials in India (15), and in 2016 it was noted that a clinician had simultaneously run 10–15 (16). We were interested in how many trials PIs in India tend to run at a time. Here, we performed a comprehensive study to determine how many interventional trials of certain types a given PI had run in India. For the PIs with the largest number, we wished to determine how many studies each of them had run in a given year.

## Materials and methods

We summarize the methodology here, with further details in Supplementary material 1 and the files referenced therein, that is, Supplementary materials 2–10. Henceforth, we use the words “trial” and “study” interchangeably.

From 26 to 28 April 2023 we accessed the trial records then hosted by CTRI. We wrote a script in R (available in Supplementary material 1) that was used to download all 52,149 records [which are available at (17)] and then to scrape the required data (Supplementary material 2). The script is available in Supplementary material 1. The data in Supplementary materials 3–8 were generated by Excel functions. Each data point was extracted by two authors, independently. The corresponding author was involved in each step of the study, verifying the data and sorting out any doubts in discussion with the other authors. All field names mentioned below are italicized.

**Figure 1:**
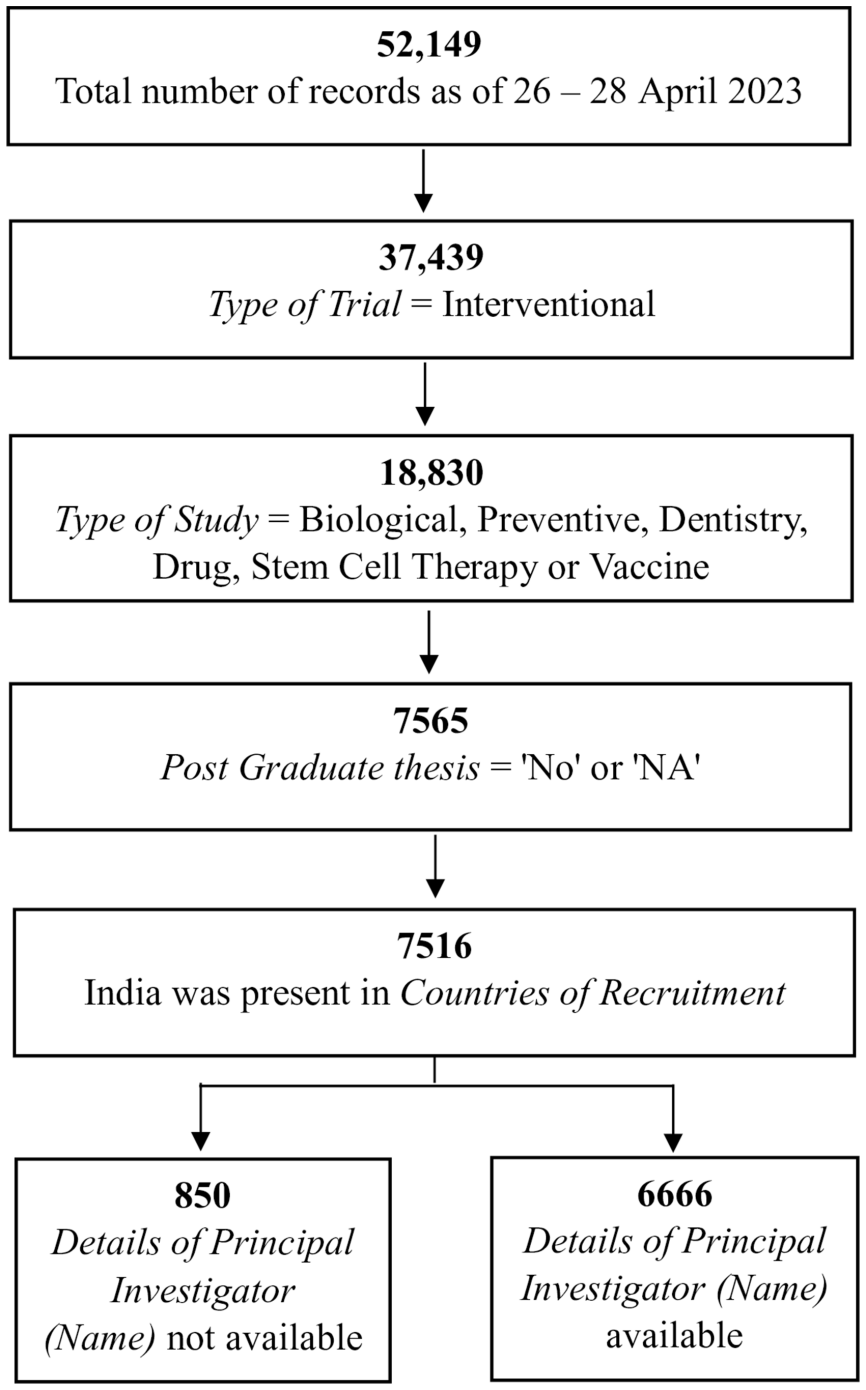
The steps used to identify the 6,666 records of interest.

Of the 52,149 records, we shortlisted those in which (a) the *Type of Trial* was “Interventional” (Supplementary material 2); (b) the *Type of Study* was “Biological,” “Preventive,” “Dentistry,” “Drug,” “Stem Cell Therapy,” or “Vaccine” (Supplementary material 2); (c) records in which the *Post Graduate Thesis* was “No” or “NA” (Supplementary material 3); and (d) those in which “*Countries of Recruitment*” mentioned India (Supplementary material 3). Of the 7,516 records shortlisted, there was no information in *Details of Principal Investigator or overall Trial Coordinator (multi-center study)* for 850 records, leaving 6,666 records that were taken forward (Supplementary material 4). The filtering of records up to this stage is shown in Figure 1.

**Figure 2:**
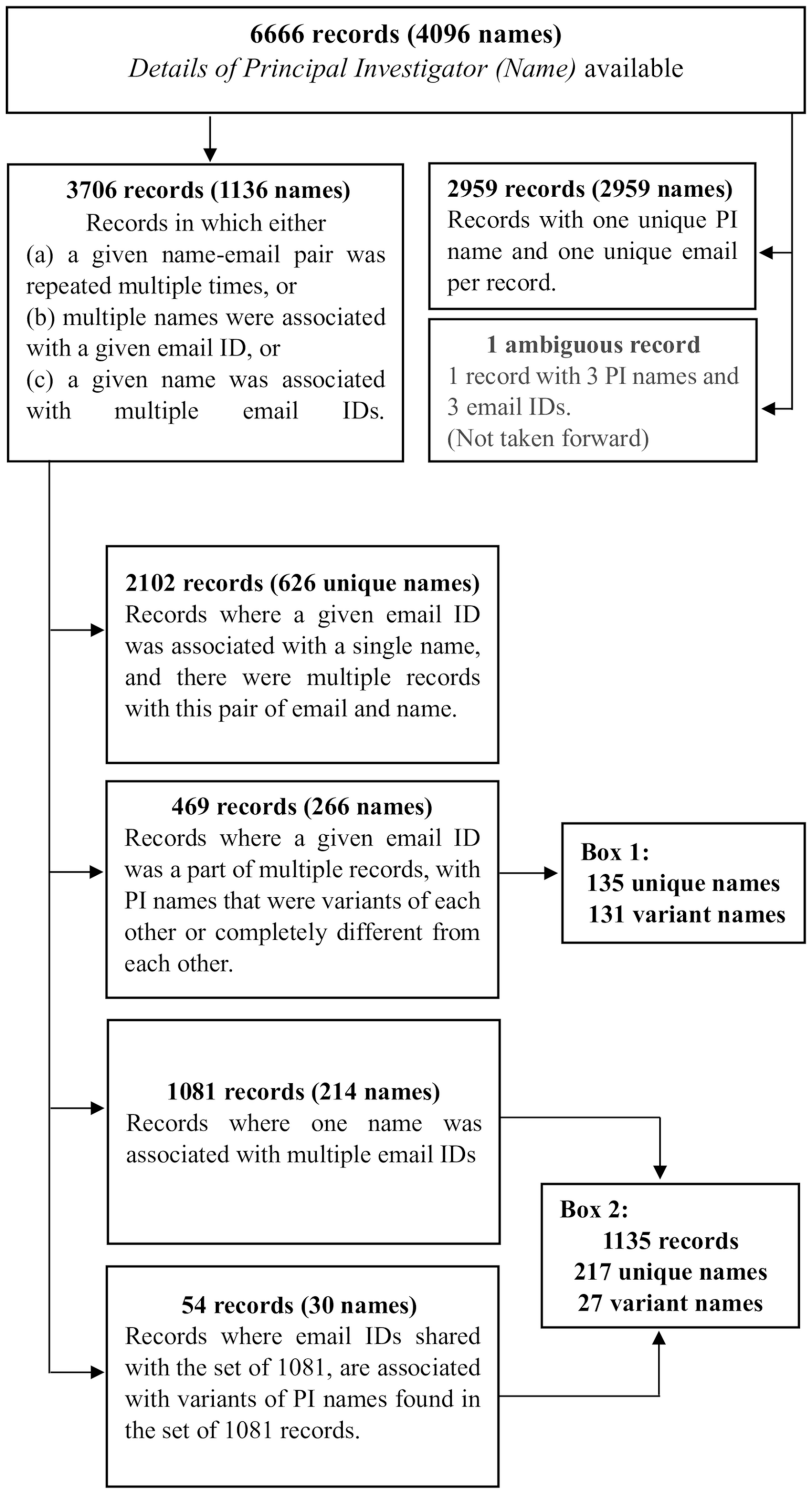
The first steps in processing the 6,666 records. In order to identify the list of unique names of PIs that had run these trials, in the first step, the 6,666 records were categorized into three sets of 3,706 records (1,136 names), 2,959 records (2,959 names) and 1 record (3 names). The 3,706 records were processed further in this figure. The information in all boxes, except the “variant names” in Boxes 1 and 2, are detailed in Supplementary materials 4, 5. Boxes 1 and 2 are described subsequently in Supplementary material 9.

**Figure 3:**
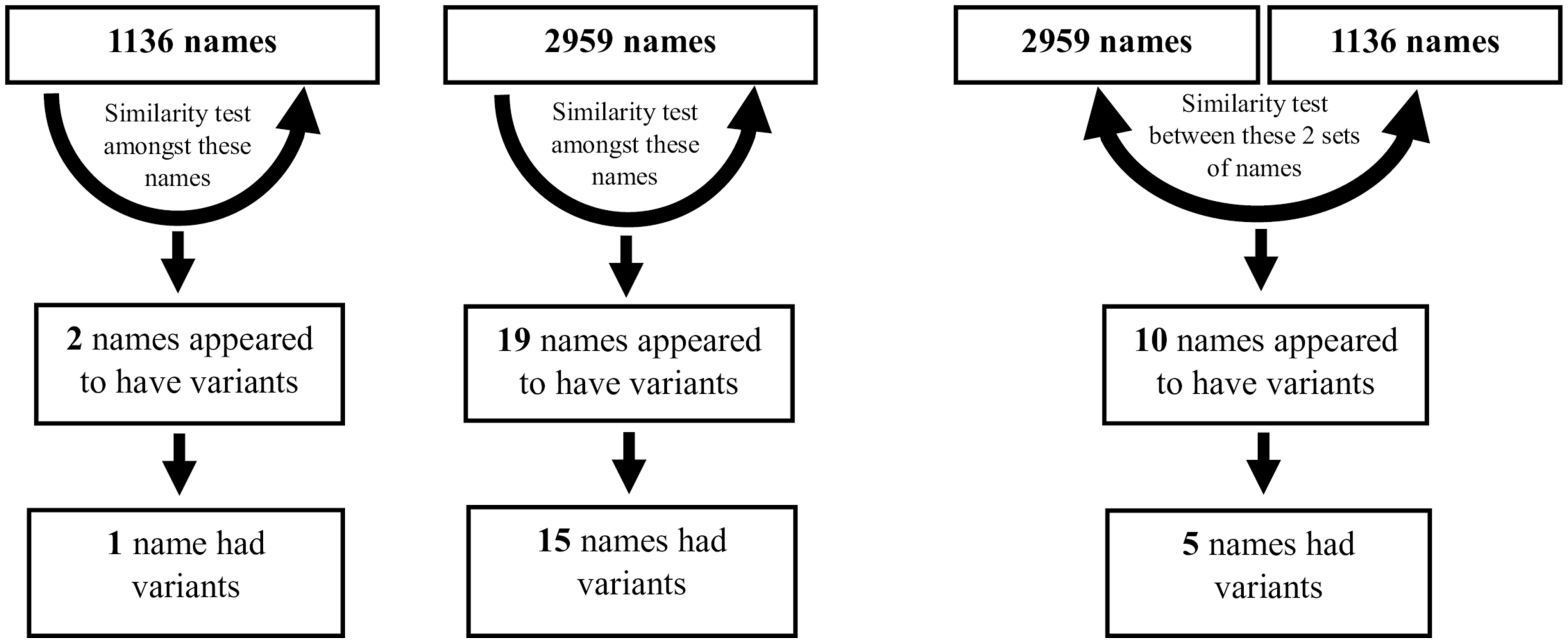
Two similarity matrix methods were used on the 1,136 and 2,959 sets of names individually, and on the two sets together, in order to identify variant names. Further details are in Supplementary material 9.

**Figure 4:**
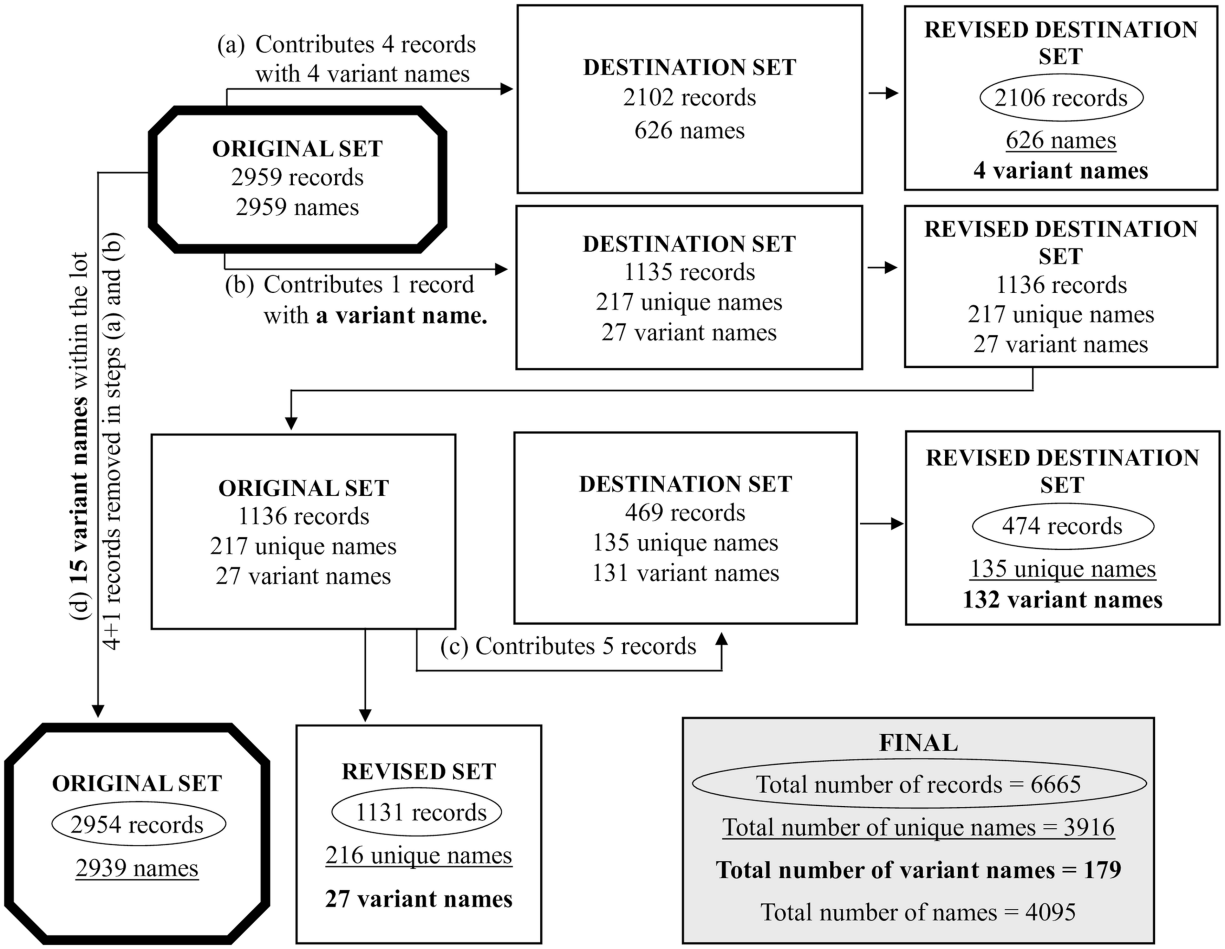
The redistribution of some records between sets of trials, after running the two similarity matrix methods. As depicted in Figure 2, the 6,666 records were distributed to the sets of 3,706, 2,959 and 1 record(s). As described in Supplementary material 4, the single record was set aside and not processed further. This reduces the 6,666 records to 6,665 records. The set of 3,706 records was further distributed into 2,102, 469, 1,081 and 54 records (with the latter two combined as 1,135 records). Due to the use of the two similarity matrix methods, some records transferred from one set to another because of name variants. As such: (a) the set of 2,959 records donated 4 records to the 2,102 records of Figure 2; (b) the set of 2,959 records donated 1 record to the 1,135 records of Figure 2; (c) the set of 1,135 records [1,136 after step (b)] donated 5 records to the 469 records of Figure 2; (d) the set of 2,959 records had donated 4 records and 1 records, as mentioned in (a) and (b), reducing it to 2,954 records. Due to 15 name variants within the set of 2,954, the total number of names was reduced to 2,939. In this figure, the two thick bordered octagons, represent the processing of 2,959 records. From the various steps, we obtain the circled numbers that indicate the unique number of records, the underlined numbers that indicate the unique names, and the numbers in bold that indicate the name variants. The box labeled FINAL has the overall list of records, unique names, name variants and names. Further details are in Supplementary materials 9, 10.

We wished to know how many individuals acted as Principal Investigators and which of them had run the most trials. For this, we had to identify the list of unique PIs, accounting for variations in a given name. That is, did one name always represent the same person and did two names sometimes correspond to the same PI? For this, we examined the field *Details of Principal Investigator or overall Trial Coordinator (multi-center study).* After setting aside one record in which the PI could not be unambiguously determined, we were left with 6,665 records. The various steps in the processing of these 6,665 records are outlined in Figures 2–4 and detailed in Supplementary materials 4–10.

We then examined the distribution of the number of trials run per PI.

## Results

A summary of the results is available at each step of the expanded methodology, detailed in Supplementary material 1 and in the other additional files referenced therein.

**Figure 5:**
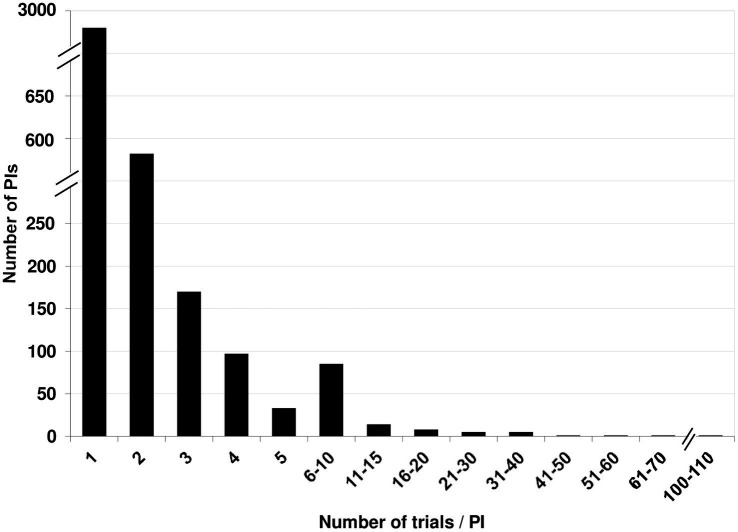
The number of PIs who ran a given number of trials vs. the number of trials per PI.

A total of 4,095 PI names were associated with these 6,665 records. 48 names (1.2%) linked to 61 records (0.91%) could not be unambiguously identified as unique individuals or as the same person already identified, and were set aside. A total of 179 were name variants, leaving us with 3,916 unique names. Of these, 2,963 (75.7%) PIs had run a single study. A mere 532 (13.6%) had run two, and 170 (4.3%) had run three trials. Only 251 (6.4%) had run more than three. There was a steady decrease in the fraction of PIs who had run more than three studies (Figure 5).

Only 14 PIs had run 20 or more trials [available at Borah et al. (17)]. These ran between 2009 and 2023, inclusive. The maximum number of trials run by a single PI was 108. Of these, the largest number was 53, run in 2022. The largest number of studies run in 1 year by a different PI was 31, in 2012. The 14 PIs were affiliated with local pharma companies (6 cases), local or global contract research organizations (4), multinational pharma companies (3), and the Central Council for Research in Homeopathy (1). The top few years in which the PI ran the maximum number of trials are presented in Table 1.

**Table 1:** The 14 PIs and the highest number of trials run by each in a given year.

		Years														
PI	Number of records	2009	10	11	12	13	14	15	16	17	18	19	20	21	22	23
																
PI 1	108													49	53	48
PI 2	61	11	19											11	11	
PI 3	53			30	31	27										
PI 4	44										7		6	13	6	
PI 5	40	6	28	14												
PI 6	34				11	6	8	6								
PI 7	34													19	23	11
PI 8	31													20	14	14
PI 9	30			10	12	8										
PI 10	29													16	17	17
PI 11	25												7	11	10	
PI 12	24		5	9	4											
PI 13	21										5	5			8	7
PI 14	21							7	11	9		7	7			

For each PI, the years in which he or she ran the three largest number of studies are presented here. In cases where more than 1 year was spent hosting the three largest number of trials, data is presented for 4 or 5 years. The PIs’ names have been replaced by numbers.

## Discussion

In 2011, it was noted that a PI had run 25 trials in India (15), although it was not clear how many of these had run simultaneously. In 2016, it was noted that a clinician had simultaneously run 10–15 trials (16). Probably those cases had come to the authors’ attention by chance, since we note that particular PIs had run a larger number of studies in those time frames. In our study, we systematically searched for PIs who had run a large number of trials, whom we call “high-burden PIs.” As such, we have outlined a methodology that will serve the field in the future as well.

We first discuss the issue of PI names that were ambiguous. Although we put in significant effort to determine whether PIs with identical or similar names were the same individual, there were cases where there was no basis to assume that this was so. Since a person may have moved, his or her email, phone, fax, affiliation, and zip code may all have changed. Further, a person’s name may have changed due to marriage, a change in gender or a change in religion, for instance. There was no way to identify such cases. For all such individuals, we may have underestimated the number of trials they had run. That being said, there were relatively few PIs who had run a large number of studies, and it is unlikely that this overall picture would change significantly even if the identification of the number of trials run by each PI was completely accurate.

Since we have touched upon methodological issues, we now come to the issue of studies that did not explicitly list a date of completion. It is well known that recruitment of participants to a trial tends to be delayed (18, 19), and these delays can be of different durations. The *Date of First Enrollment* and the *Estimated Duration of Trial* were usually listed in the CTRI record, but the *Date of Study Completion* may not have been. Therefore, in some cases, we had to estimate the *Date of Study Completion*. We had no way to determine whether or not a given study was delayed, and if so, by how much. We took a conservative approach and merely added 3 months to the *Estimated Duration of Trial* of each study for which the *Date of Study Completion* was not provided, in order to arrive at the estimated *Date of Study Completion*. We believe that this probably underestimates the duration of some trials. Were the actual *Date of Study Completion* to be available, the number of studies run in a given year by some PIs, including some high-burden PIs, may have been found to be even higher that what we report.

To come to our findings: At one point, the Government of India had mandated the rule of three. It is unclear why the number three was chosen and why it wasn’t a rule of two, four or some other number. Regardless, the purpose of restricting the number of trials per PI at a given time was undoubtedly to ensure that the highest levels of participant safety, data quality, research outcome reliability and ethical standards were maintained.

Since the rule did exist for a while, let us examine our results with reference to it. We have found that most PIs ran very few trials, and only 251 (6.4%) had run more than three studies over the years. As such, most PIs had abided by the rule of three, even though it was not in force for most of the years under consideration. Although we wished to understand how many PIs had run “too many” trials, we felt that it would be a large, and largely unnecessary, task to determine how many PIs had run more than three studies in a given year. Instead, our focus was on the PIs who ran the most trials. We found that one PI had run 108 trials from 2006 to 2023, and had run a maximum of 53 in a single year, i.e., in 2022. Several other PIs, too, had run more than the “10–15” studies in a given year, a figure that was considered unacceptably high in a 2016 report (16).

One needs to be concerned about a given PI running many trials in India. It is known that the specialist-patient ratio in the country is extremely poor (16), and it is also widely acknowledged that the workload on Indian doctors can be extremely heavy. This gives rise to the apprehension that a doctor may not be able to take adequate care of the participants in multiple ongoing studies. However, all the high-burden PIs, with one exception, worked for either Indian or multinational pharmaceutical companies or contract research organizations. The excess of trials under the supervision of a single Principal Investigator in the pharmaceutical development area can be attributed to several factors. Pharmaceutical companies often have more substantial financial resources than academic institutions, allowing them to support multiple simultaneous trials under a single PI. These resources enable comprehensive administrative and logistical support, which helps manage multiple projects efficiently. The pharmaceutical industry is highly competitive and fast-paced, with a strong emphasis on rapidly bringing new drugs to market. This urgency can lead to a concentration of trials under experienced PIs who are trusted to deliver reliable and timely results. Industry PIs often have specialized expertise and a proven track record in conducting clinical trials, making them valuable assets for overseeing multiple studies. In contrast, academic settings may have a more diverse range of research interests and limited funding, which can diffuse focus and resources.

Being a PI in the pharmaceutical industry or clinical research organizations (CROs) can potentially offer more flexibility compared to those in academia or practicing physicians. This is because industry-based PIs often focus solely on research and clinical trials management, without the direct patient care responsibilities that practicing physicians have. This specialization allows industry-based PIs to dedicate more time and effort to the regulatory and administrative aspects of clinical trials, ensuring compliance with protocols and regulatory requirements. However, the specific degree of flexibility can vary depending on the organization and the specific regulatory environment governing clinical trials in India. We are not aware of any commentary or study about this issue in the Indian context.

Most likely the high-burden PIs were not involved in regular patient care. Therefore, whereas the issue of regular patient care may not have compounded the care of participants in trials for some or all of the high-burden PIs, this situation existed even when the rule of three was in force. That is, the rule of three was imposed irrespective of whether or not a PI was involved in regular patient care. The government must have had its reasons for this rule. Therefore, although all of the high-burden PIs may have run their trials perfectly, any plan to audit studies in India should include such cases.

We reiterate that since the enactment of the New Drugs and Clinical Trials Act, 2019 (20), there is no legal limit on the number of trials a given PI can run simultaneously. This lack of legal restriction means that PIs in the pharmaceutical industry can oversee numerous trials at once. However, this practice raises concerns about how unmonitored or unregulated activities might increase risks to participants, highlighting the need for stringent oversight to ensure participant safety and trial integrity.

We note that CTRI has many strengths. A CTRI record has 41 fields, providing rich data on many aspects of a trial. Nevertheless, it also has occasional weaknesses such as incomplete or internally inconsistent data (20), the lack of a detailed audit trail for each record (21), ambiguity in which Ethics Committees is linked to particular site (22) and the non-standard classification of sponsors (23).

Based on this study, we make the following recommendations: (a) since multiple versions of a PI’s name may be used, systems need to be implemented in CTRI to prevent this. Each PI name should be connected to a unique ID that does not change with time, like the ORCID ID (24) used to identify researchers with academic publications, or the BIORAPP ID issued by the Government of India for particular types of research proposals that may require clearance from multiple agencies (25). (b) Since some PIs have run a large number of trials, the IECs need to be cautious while approving proposed studies. While registering a trial with CTRI, if a sponsor is required to state how many studies the PI is currently running, this transparency is likely to make the IECs more cautious in approving proposals. (c) Conceivably, there could be a link from each PI’s name to a list of his or her other trials, and the state of completion of those trials. (d) Conceivably, CTRI should flag each record that pertains to a Phase III study that is critical to a particular drug’s development, for instance. CTRI could flag such trials, based on sponsor submissions of relevant information. This would enhance transparency. (e) The government needs to consider whether a cap, perhaps not as restrictive as the rule of three, is required, given the very large number of trials that a handful of PIs are now running. Undoubtedly various factors influence the number of trials that a PI can handle well at a given time, including trial complexity, resources available, PI capacity, study duration, and study completion or termination rates. Some discussion is probably required to come to a more nuanced limit. For instance, regulations may mandate that certain support structures be in place before a PI can run more than a certain number of trials at a time. (f) The regulator should monitor the number of studies being run by a given PI more actively. (g) CTRI should mandate the updating of information on the status of a given clinical trial, until it is completed and the results made public. This should be yearly or more frequently if possible. The United States registry ClinicalTrials.gov mandates a yearly update until the study is completed and results made public (26).

Finally, we note that the study has some limitations, as follows:

- Although we selected *Type of Trial* as “Interventional,” it is possible that some interventional trials were not labeled as such and therefore that we missed them. Likewise, we selected a subset of *Types of Study.* It is possible both that we missed some relevant trials due to mislabeling and also that some of the PIs of this work carried out some other interventional studies that we did not select. If we missed cases due to our selections of the *Type of Trial* and *Type of Study*, then the number of studies per person in a given year that we report is lower than what it actually was.
- We have no way to validate the data in a given CTRI record. We are aware of many categories of errors or ambiguities in CTRI (21, 22, 27, 28), as reported for other registries as well (29–32), but have assumed that the name of the PI, the start date of the trial, its estimated duration, and the date of completion of the study, where available, are correct. If any of these is incorrect, then our analysis is correspondingly incorrect.
- The large number of CTRI records with incomplete data, especially with regard to the date of completion, meant that we had to estimate the date of completion in those cases. Since we were extremely conservative in providing only a 3-month delay beyond the expected date of completion, it is likely that several PIs actually ran trials for longer than what we calculated. This would have lead to an increase in the number of studies running in a given year for some PIs, including some high-burden PIs. If so, the number of trials per person in a given year that we report is lower than what it actually was.
- As noted above, we may have underestimated the number of trials run by a given individual in case the name was identical or similar to another name, with no common identification information, or in case the person had changed his or her name.
- There were three instances in which the trial start date was absent or its estimated duration was marked zero, that were excluded from the Gantt chart creation process. So we know that the number of trials reported per person in a given year has been underestimated in this minuscule number of cases.

In summary, we were able to identify 3,916 unique names that were linked to particular categories of interventional trials, totaling 6,665 studies, registered with CTRI. Of these, 2,963 (75.7%) PIs had run a single trial, and only 251 (6.4%) had run more than three. Only 14 PIs (0.36%) had run 20 or more trials, between 2009 and 2023. The 14 PIs were affiliated with local pharma companies (6), local or global contract research organizations (4), multinational pharma companies (3), and the Central Council for Research in Homeopathy (1). The maximum number of trials run by a single PI was 108. Of these, 53 were run in 1 year, 2022. Although the low number of studies run by most PIs is acceptable, the large number run by a few PIs is of concern. We suggest ways to prevent this from happening in the future. Also, other countries may wish to carry out a similar analysis to obtain a detailed picture of how many trials are run by each of their PIs in case there is cause for concern.

## Data availability statement

The original contributions presented in the study are included in the article/Supplementary material, further inquiries can be directed to the corresponding author.

## Author contributions

RB: Formal analysis, Investigation, Methodology, Validation, Writing – review & editing. AS: Formal analysis, Investigation, Methodology, Validation, Writing – review & editing. KR: Formal analysis, Investigation, Methodology, Validation, Writing – review & editing. VV: Methodology, Writing – original draft, Writing – review & editing. GS: Conceptualization, Formal analysis, Funding acquisition, Investigation, Methodology, Project administration, Resources, Supervision, Validation, Writing – original draft, Writing – review & editing.
