# Supplementary material for: In India, most principal investigators have run very few trials over the years
Source: Front Med (Lausanne). 2024 Aug 7;11:1424570. doi: 10.3389/fmed.2024.1424570 (PMC11335508; doi:10.3389/fmed.2024.1424570)
Supplement: Supplementary file 1 [file Table_1.DOC]

**Additional file 1. Expanded methodology and a summary of the results at each step**

**1. Downloading records from the CTRI database and web scraping the required data**

The records in CTRI were downloaded from <https://ctri.nic.in/Clinicaltrials/login.php?id>=

between 26–28 April 2023 using Script 1 (written in R), which is available, along with the other scripts (all written in Python), later in this file. A timestamp file was also created to record the date and time of each downloaded page. All 52,149 records were downloaded and are available at https://dx.doi.org/10.17605/OSF.IO/Y7CM2.

We web-scraped the 52,149 records using Script 2 (Additional file 2) for the fields *Type of Trial* and *Type of Study*. Only 37,439 records were retained in which *Type of Trial* was ‘Interventional’, and 18,830 records where *Type of Study* was 'Biological', 'Preventive', 'Dentistry', 'Drug', 'Stem Cell Therapy' or 'Vaccine'. The output of these steps is available in Additional file 2.

We web-scraped these 18,830 records using Scripts 3a and 3b for the fields *Post Graduate Thesis* and *Countries of Recruitment*. Only 7565 records were retained where *Post Graduate Thesis* was ‘No’ or ‘NA’, and 7516 where the *Countries of Recruitment* field contained ‘India’. The output of these steps is available in Additional file 3. The filtering of records up to this stage is captured in Figure 1.

**2. Analyzing the 7516 records to find the unique number of trials per Principal Investigator (PI)**

From these 7516 records, 850 records were filtered out because the sub–field *Name* of the field *Details of Principal Investigator or overall Trial Coordinator (multi-center study*) was ‘-’, ‘NA’, ‘Nil’ or ‘Not applicable’. This left 6666 records to be processed. One record was not taken forward due to ambiguous information, leaving 6665 records to be processed. The filtering of records up to this stage is also captured in Figure 1.

Any white-space characters, special characters, salutations, degrees, or other designations were removed from the subfield *Name* in the 6665 records.

These 6665 records, comprising 4095 names, some of which were name variants, were categorized into two sets as follows. We have also devised a notation of an ‘X/Y/Z set’ where X = number of records, Y = number of unique names, and Z = number of name variants (where applicable).

- 2959 records (2959 names), i.e., the 2959/2959 set: Cases of a single occurrence of a PI name with its associated email ID.
- 3706 records (1136 names), i.e., the 3706/1136 set: In which

(a) a given PI name-email pair was repeated across records,

(b) multiple PI names were associated with a given email ID across records, or

(c) one PI name was associated with different email IDs across records.

The output of this step is available in Additional file 4 and in Figure 2.

The 3706/1136 and 2959/2959 sets were taken forward and analyzed separately. The analysis of the 3706 records is available in Additional files 5–10 and also captured in Figures 2–4, and that of the 2959 records in Additional files 9–10 and in Figures 3–4.

**3. Analyzing the 3706/1136 set**

In order to determine whether one name always represented the same PI and whether two names corresponded to the same PI, we first used emails as a method of identification. The 3706/1136 set was divided into the following four sets (as captured in Additional file 5 and Figure 2):

1. 2102 records (626 unique names), i.e., the 2102/626 set:
   The set of 2102 records had 626 unique names. A given email ID was associated with a single PI name, and there were multiple records with this pair of name and email.
2. 469 records (266 names, of which 135 were unique and 131 were name variants), i.e., the 469/135/131 set:
   In the set of 469 records, a given email ID was part of multiple records, and linked to PI names that were the same, were variants of each other, or were completely different from each other. There were a total of 135 unique names and 131 name variants among the 469 records (Box 1 of Figure 2).
3. 1081 records (214 names), i.e., the 1081/214 set:

In the set of 1081 records, a single name was linked to multiple email IDs across records.

1. 54 records (30 names), i.e., the 54/30 set:
   54 records had email IDs that were a subset of the 1081 records of step iii above, but were linked to variations of PI names found in the set of 1081 records.
2. 1135 records (with 217 unique names and 27 variants), i.e., the 1135/217/27 set:
   Combining the 1081 and 54 sets (of steps iii and iv, above) yielded 1135 records. There were a total of 217 unique names and 27 name variants among the 1135 records (Box 2 of Figure 2).

After using emails as a mode of identification, we went on to use phone numbers, affiliations, fax numbers, or zip codes in order to determine whether one name always represented the same PI and whether two names corresponded to the same PI. These steps are captured in Additional files 6–8.

**4. Execution of the two similarity matrix programmes**

Although we used email, phone numbers, etc. to determine whether two names corresponded to the same PI, and whether one name always represented the same PI, name variants complicated the matter. A given name may have had what appeared to be variants. In order to identify name variants that represented the same person, two similarity matrix programmes were used. These were the ‘Levenshtein distance’ programme and the ‘partial ratio’ programme, both described below. The two programmes were executed utilizing Scripts 4a–4d.

(a) The Levenshtein distance programme (Script 4a) compared two strings of information and provided a score based on how few changes were required to change one string to the other. For example, the Levenshtein score obtained by comparing AAbhinav and AAbhinav was 100. That obtained by comparing AAbhinav and AbhinavDeshpande was 58.33. We considered 80% or more as a good score. We shortlisted cases with a score of 80% or more using Script 4b and took them forward for manual investigation.

(b) The partial ratio programme (Script 4c) used a variation of the Levenshtein distance algorithm. It calculated the similarity score by comparing the shorter string against all the substrings of the longer string. This method generated many false positive results, and hence the cut-off score was raised to 90%. For example, the score obtained by comparing AAbhinav and AAbhinav was 100, whereas the score obtained by comparing AAbhinav and AbhinavDeshpande was 93.33. We shortlisted cases with a score of 90% or more using Script 4d and took them forward for manual investigation.

These programmes were used on the 3706/1136 and 2959/2959 sets individually, and in a comparison of the two. For the 3706/1136 set, the analysis was carried out on the 2102/626, 469/135/131, and 1135/217/27 subsets mentioned in steps i, ii and v above.

The cases shortlisted by the two methods were collated and de-duplicated. Each case was manually checked, including by google searches for confirmation of whether or not two names were the same person. We found that some sets had variants of names found in other sets. These had to be reconciled, as described here, and as captured in Figure 4:

1. From the larger 2959/2959 set, 4 records were transferred to the 2102/626 set, because the larger set contained 4 name variants. For example, there were a total of 4 occurrences of the name variants DRaghunadharao and Raghunadharao. As such, the 2102/626 set became 2106/626/4.
2. From the larger 2959/2959 set, 1 record was transferred to the 1135/217/27 set, because the larger set contained 1 name variant that was already present in the 1135/217/27 set. As such, the 1135/217/27 set became 1136/217/27.
3. From the larger 2959/2959 set, 4+1 records had been transferred, as mentioned in (a) and (b) above. There were also 15 name variants within the 2959/2959 set. Overall, the 2959/2959 set was reduced to 2954/2939.
4. From the larger 1136/217/27 set, 5 records were transferred to the 469/135/131 set, because the larger set contained 5 records with a name common to the 469/135/131 set, and also one new name variant. As such, the 469/135/131 set became 474/135/132.
5. The larger set of 1136/217/27, after donating 5 records to the 469/135/131 set, became 1131/216/27.

In summary, the 6665 records contained 4095 names, which were the sum of 3916 unique names and 179 name variants.

The output of these steps is available in Additional files 9–10.

**5. PIs with 20 or More Trials: Analysis of the number of trials per PI and the duration of each.**

Fourteen PIs had conducted 20 or more trials over the years. We analyzed the number and duration of these trials.

We prepared a ‘Gantt chart’ reflecting which years a given PI’s trials had run in. In order to do this, we used Script 5 to obtain the start and end dates of each trial run by a particular PI. If the end date was not specified, we added three months to the *Estimated duration of trial*. For example, if (a) the start date was ‘11-01-12’ (in dd-mm-yy format), (b) the estimated duration of the trial was 12 months, and (c) the end date was unspecified, we estimated the end date to be ‘11-04-13’. To be noted, a total of three instances where the trial start date was absent or the *Estimated duration of trial* was marked 0 were excluded from the Gantt chart creation process. If a trial ran for more than one calendar year, it would be marked for each year in the chart. As such, for the example mentioned above, the trial was marked as having run in both 2012 and 2013.

For each of the 14 PIs, an Excel file was prepared. The information captured in different sheets was: (i) details of each trial, such as trial registration number, *Date of registration*, *Date of first enrollment (India)*, *Date of study completion (India)*, *Estimated duration of trial*, and *Recruitment status of trial (India)*. If required, three months were added to arrive at an estimated *Date of study completion (India)*. One file was created per PI, with the numbered PI in the file name. This file contains (i) a summary Gantt chart reflecting the years in which the PI’s trials had run; (ii) the finalized list of start and stop dates for each trial; and (iii) details of each trial. These 14 files are available as Additional file 11 at https://dx.doi.org/10.17605/OSF.IO/Y7CM2.

For each PI, we identified the years in which the maximum number of trials were conducted. The top three years were captured in Table 1. If two or more years had these ‘maximum number of trials’, then four or five years were listed in place of the top three years.

**Scripts 1–5 used in this work:**

#************************************Script - 1***************************

#This code was utilised for downloading all the records present in the CTRI database.

libraries = c( "XML","robotstxt", "tidyft","data.table", "DBI", "httr", "RSQLite","tidyverse","rvest","stringr","robotstxt","selectr","xml2","dplyr","forcats","magrittr","tidyr","ggplot2","lubridate","tibble","purrr","googleLanguageR","cld2")

lapply(libraries, require, character.only = TRUE)

counter=0

new_function <- function(a) {

if (length(a) == 0) {

a <- "NA"

} else if (a == "") {

a <- "NA"

} else {

return(a)

}

}

#-- Step1: Downloading all the CTRI Webpages in HTML.

ids = c(1:160000)

for (i in seq_along(ids)) {

myurl = paste0("http://ctri.nic.in/Clinicaltrials/pmaindet2.php?trialid=", ids[i])

url = url(paste0("http://ctri.nic.in/Clinicaltrials/pmaindet2.php?trialid=",ids[i]))

ctri_page = read_html(url)

keyword = ctri_page %>% html_nodes("td") %>% html_text() %>% str_extract("Invalid Request")

keyword = toString(keyword)

##This is done because there are many records which have their particular links, but no content is present in those records. Instead it is displayed as "Invalid Request".

if (keyword != "Invalid Request") {

myfile = paste0("ctri_page",ids[i],".html")

download.file(myurl, destfile = myfile, quiet = TRUE)

time_of_download = as.character(timestamp())

reg_num <- ctri_page %>% html_nodes("td tr:nth-child(1) td+ td > b") %>% html_text() %>% str_squish() %>% str_trim()

reg_num <- toString(reg_num)

reg_num <- new_function(reg_num)

time_stamp = data.frame(Trial_ID = as.character(ids[i]),

downloaded_time = time_of_download,

URL = as.character(myurl),reg_num)

write.table(time_stamp, "time_stamp_ctri.csv", sep = ",",row.names = FALSE, col.names = !file.exists("time_stamp_ctri.csv"), append = T)

counter = counter + 1

print(paste("Count = ", counter,"ID = ",ids[i]))

}

else {

file <- data.frame(Trial_ID=as.character(ids[i]),URL=as.character(myurl))

write.table(file, "time_stamp_ctri_invalid.csv", sep = ",",row.names = FALSE, col.names = !file.exists("time_stamp_ctri_invalid.csv"), append = T)

print(paste("Count=",counter,"ID = ",ids[i],"but page is invalid one"))

}

}

#****************************Script - 2***********************************

#This script was used for web-scraping the field 'Type of Trial' and 'Type of Study' from all the downloaded records

libraries = c( "XML", "tidyft","data.table", "DBI", "httr", "RSQLite","tidyverse","rvest","stringr","robotstxt","selectr","xml2","dplyr","forcats","magrittr","tidyr","ggplot2","lubridate","tibble","purrr","googleLanguageR","cld2")

lapply(libraries, require, character.only = TRUE)

ids <- c(1:84639)

counter = 0

for (i in seq_along(ids)) {

myfile = paste0("ctri_page",ids[i],".html")

if (file.exists(myfile)) {

ctri_page <- read_html(myfile)

if(is_empty(ctri_page)) {

next

}

new_function <- function(a) {

if (length(a) == 0) {

a <- "NA"

} else if (a == "") {

a <- "NA"

} else {

return(a)

}

}

Trial_ID <- ids[i]

Registration_number <- ctri_page %>% html_nodes("td tr:nth-child(1) td+ td > b") %>% html_text() %>% str_remove_all('\"') %>% toString() %>% str_trim() %>% str_squish()

Registration_number <- new_function(Registration_number)

Date_of_registration <- ctri_page %>% html_nodes("td+ td") %>% .[[1]] %>% html_text() %>% str_remove_all('\"') %>% toString() %>% str_trim() %>% str_squish()

Date_of_registration <- new_function(Date_of_registration)

Last_modified_on <- ctri_page %>% html_nodes("td+ td") %>% .[[2]] %>% html_text() %>% str_remove_all('\"') %>% toString() %>% str_trim() %>% str_squish()

Last_modified_on <- new_function(Last_modified_on)

Public_title_of_study <- ctri_page %>% html_nodes("td+ td") %>% .[[7]] %>% html_text() %>% str_remove_all('\"') %>% toString() %>% str_trim() %>% str_squish()

Public_title_of_study <- new_function(Public_title_of_study)

Scientific_title_of_study <- ctri_page %>% html_nodes("td+ td") %>% .[[8]] %>% html_text() %>% str_remove_all('\"') %>% toString() %>% str_trim() %>% str_squish()

Scientific_title_of_study <- new_function(Scientific_title_of_study)

Type_of_trial <- ctri_page %>% html_nodes("td+ td") %>% .[[4]] %>% html_text() %>% str_remove_all('\"') %>% toString() %>% str_trim() %>% str_squish()

Type_of_trial <- new_function(Type_of_trial)

Type_of_study <- ctri_page %>% html_nodes("td+ td") %>% .[[5]] %>% html_text() %>% str_remove_all('\"') %>% toString() %>% str_trim() %>% str_squish()

Type_of_study <- new_function(Type_of_study)

Type_of_study2 <- ctri_page %>% html_nodes("td+ td") %>% .[5]

Type_of_study2 <- new_function(Type_of_study2)

file <- data.frame(Trial_ID,Registration_number,Date_of_registration,Last_modified_on,Public_title_of_study,Scientific_title_of_study,Type_of_trial,Type_of_study,Type_of_study2)

write.table(file,"RB_scrape_type_of_trial_type_study2.csv", sep = ",",row.names = FALSE, col.names = !file.exists("RB_scrape_type_of_trial_type_study2.csv"), append = T)

counter = counter + 1

print(paste("Count = ", counter,"ID = ",ids[i]))

}

}

#********************************Script - 3****************************

#These scripts 3a-3b were used for web-scraping the field 'Post Graduate Thesis','Countries of Recruitment', and 'Details of Principal Investigator or #overall Trial Coordinator (multi-center study)'

#Script - 3a

libraries = c( "XML", "tidyft","data.table", "DBI", "httr", "RSQLite","tidyverse","rvest","stringr","robotstxt","selectr","xml2","dplyr","forcats","magrittr","tidyr","ggplot2","lubridate","tibble","purrr","googleLanguageR","cld2")

lapply(libraries, require, character.only = TRUE)

ids <- data.frame()

ids <- read.csv("18830_ids.csv")

colnames(ids) <- "ids"

counter=0

for (row in 1:nrow(ids)) {

i <- ids[row,1]

myfile = paste0("ctri_page",i,".html")

if (file.exists(myfile)) {

ctri_page <- read_html(myfile)

if(is_empty(ctri_page)) {

next

}

new_function <- function(a) {

if (length(a) == 0) {

a <- "NA"

} else if (a == "") {

a <- "NA"

} else {

return(a)

}

}

Trial_ID <- i

Registration_number <- ctri_page %>% html_nodes("td tr:nth-child(1) td+ td > b") %>% html_text() %>% str_remove_all('\"') %>% toString() %>% str_trim() %>% str_squish()

Registration_number <- new_function(Registration_number)

Post_graduate_thesis <- ctri_page %>% html_nodes("td+ td") %>% .[[3]] %>% html_text() %>% str_remove_all('\"') %>% toString() %>% str_trim() %>% str_squish()

Post_graduate_thesis <- new_function(Post_graduate_thesis)

for (num in 1:5186){

COR_label1 <- ctri_page %>% html_nodes("td") %>% .[[num]] %>% html_text() %>% str_remove_all('\"') %>% str_remove_all("\n") %>% str_remove_all("\r") %>% str_remove_all("\t") %>% toString() %>% str_trim() %>% str_squish()

if ((COR_label1 == "Countries of Recruitment") || (COR_label1 == "Countries of Recruitment Modification(s)"))

{

Countries_of_recruitment <- ctri_page %>% html_nodes("td") %>% .[[num+1]] %>% html_text() %>% str_remove_all('\"') %>% str_remove_all("\n") %>% str_remove_all("\t") %>% str_remove_all("\r") %>% toString() %>% str_trim() %>% str_squish()

COR_label <- ctri_page %>% html_nodes("td") %>% .[[num]] %>% html_text() %>% str_remove_all('\"') %>% str_remove_all("\n") %>% str_remove_all("\r") %>% str_remove_all("\t") %>% toString() %>% str_trim() %>% str_squish()

selector_number <- num

break

} else {

Countries_of_recruitment <- "Not a country name"

COR_label <- "Not a country label"

selector_number <- "Not within limits"

}

}

file <- data.frame(Trial_ID,Registration_number,Post_graduate_thesis,Countries_of_recruitment,COR_label,selector_number)

write.table(file,"RB_scrape_PG_COUNTRIES_FINAL_91_100_part2.csv", sep = ",",row.names = FALSE, col.names = !file.exists("RB_scrape_PG_COUNTRIES_FINAL_91_100_part2.csv"), append = T)

counter = counter + 1

print(paste("Count = ", counter,"ID = ",i))

}

}

#Script - 3b

libraries = c( "XML", "tidyft","data.table", "DBI", "httr", "RSQLite","tidyverse","rvest","stringr","robotstxt","selectr","xml2","dplyr","forcats","magrittr","tidyr","ggplot2","lubridate","tibble","purrr","googleLanguageR","cld2")

lapply(libraries, require, character.only = TRUE)

ids <- data.frame()

ids <- read.csv("7516_ids.csv")

colnames(ids) <- "ids"

counter=0

for (row in 1:nrow(ids)) {

i <- ids[row,1]

myfile = paste0("ctri_page",i,".html")

if (file.exists(myfile)) {

ctri_page <- read_html(myfile)

if(is_empty(ctri_page)) {

next

}

new_function <- function(a) {

if (length(a) == 0) {

a <- "NA"

} else if (a == "") {

a <- "NA"

} else {

return(a)

}

}

var <- "delimiter"

Trial_ID <- i

Registration_number <- ctri_page %>% html_nodes("td tr:nth-child(1) td+ td > b") %>% html_text() %>% str_remove_all('\"') %>% str_remove_all("\r") %>% str_remove_all("\t") %>% str_remove_all("\n") %>% toString() %>% str_trim() %>% str_squish()

Registration_number <- new_function(Registration_number)

PI_Name <- ctri_page %>% html_nodes("tr:nth-child(11) tr:nth-child(1) td+ td") %>% html_text() %>% str_remove_all('\"') %>% str_remove_all("\r") %>% str_remove_all("\t") %>% str_remove_all("\n") %>% toString() %>% str_trim() %>% str_squish()

PI_Name <- new_function(PI_Name)

PI_Designation <- ctri_page %>% html_nodes("tr:nth-child(11) tr:nth-child(2) td+ td") %>% html_text() %>% str_remove_all('\"') %>% str_remove_all("\r") %>% str_remove_all("\t") %>% str_remove_all("\n") %>% toString() %>% str_trim() %>% str_squish()

PI_Designation <- new_function(PI_Designation)

PI_Affiliation <- ctri_page %>% html_nodes("tr:nth-child(11) tr:nth-child(3) td+ td") %>% html_text() %>% str_remove_all('\"') %>% str_remove_all("\r") %>% str_remove_all("\t") %>% str_remove_all("\n") %>% toString() %>% str_trim() %>% str_squish()

PI_Affiliation <- new_function(PI_Affiliation)

PI_Address <- ctri_page %>% html_nodes("tr:nth-child(11) tr:nth-child(4) td+ td") %>% html_text() %>% str_remove_all('\"') %>% str_remove_all("\r") %>% str_remove_all("\t") %>% str_remove_all("\n") %>% toString() %>% str_trim() %>% str_squish()

PI_Address <- new_function(PI_Address)

PI_Phone <- ctri_page %>% html_nodes("tr:nth-child(11) tr:nth-child(5) td+ td") %>% html_text() %>% str_remove_all('\"') %>% str_remove_all("\r") %>% str_remove_all("\t") %>% str_remove_all("\n") %>% toString() %>% str_trim() %>% str_squish()

PI_Phone <- new_function(PI_Phone)

PI_Phone <- paste0(var,PI_Phone)

PI_Fax <- ctri_page %>% html_nodes("tr:nth-child(11) tr:nth-child(6) td+ td") %>% html_text() %>% str_remove_all('\"') %>% str_remove_all("\r") %>% str_remove_all("\t") %>% str_remove_all("\n") %>% toString() %>% str_trim() %>% str_squish()

PI_Fax <- new_function(PI_Fax)

PI_Fax <- paste0(var,PI_Fax)

PI_Email <- ctri_page %>% html_nodes("tr:nth-child(11) tr:nth-child(7) td+ td") %>% html_text() %>% str_remove_all('\"') %>% str_remove_all("\r") %>% str_remove_all("\t") %>% str_remove_all("\n") %>% toString() %>% str_trim() %>% str_squish()

PI_Email <- new_function(PI_Email)

num2=1

for (num1 in 3:96){

Site_PI_Name <- ctri_page %>% html_nodes(paste0("tr:nth-child(18) tr:nth-child(",num1,") td:nth-child(",num2,")")) %>% html_text() %>% str_remove_all('\"') %>% str_remove_all("\r") %>% str_remove_all("\t") %>% str_remove_all("\n") %>% toString() %>% str_trim() %>% str_squish()

Site_PI_Name <- new_function(Site_PI_Name)

Site_Name <- ctri_page %>% html_nodes(paste0("tr:nth-child(18) tr:nth-child(",num1,") td:nth-child(",num2+1,")")) %>% html_text() %>% str_remove_all('\"') %>% str_remove_all("\r") %>% str_remove_all("\t") %>% str_remove_all("\n") %>% toString() %>% str_trim() %>% str_squish()

Site_Name <- new_function(Site_Name)

Site_Address <- ctri_page %>% html_nodes(paste0("tr:nth-child(18) tr:nth-child(",num1,") td:nth-child(",num2+2,")")) %>% html_text() %>% str_remove_all('\"') %>% str_remove_all("\r") %>% str_remove_all("\t") %>% str_remove_all("\n") %>% toString() %>% str_trim() %>% str_squish()

Site_Address <- new_function(Site_Address)

Phone_tax_email <- ctri_page %>% html_nodes(paste0("tr:nth-child(18) tr:nth-child(",num1,") td:nth-child(",num2+3,")")) %>% html_text() %>% str_remove_all('\"') %>% str_remove_all("\r") %>% str_remove_all("\t") %>% str_remove_all("\n") %>% toString() %>% str_trim() %>% str_squish()

Phone_tax_email <- new_function(Phone_tax_email)

file <- data.frame(Trial_ID,Registration_number,PI_Name, PI_Designation, PI_Affiliation,PI_Address, PI_Phone, PI_Fax, PI_Email,Site_PI_Name,Site_Name,Site_Address,Phone_tax_email)

write.table(file,"RB_scrape_PI_Site_information.csv", sep = ",",row.names = FALSE, col.names = !file.exists("RB_scrape_PI_Site_information.csv"), append = T)

counter = counter + 1

print(paste("Count = ", counter,"ID = ",i))

}

}

}

#******************************Script - 4********************************

#These scripts 4a-4d were used for executing the similarity matrix programme, followed by pre-processing of the resultant files.

#The similarity matrix in our code is based on Levenshtein distance and partial matching, and it utilizes FuzzyWuzzy's `ratio` and `partial_ratio` #functions.

#script - 4a

import pandas as pd

from rapidfuzz import fuzz

# Load the Excel file into a DataFrame

df = pd.read_excel('1136vs2959.xlsx')

# Assuming your DataFrame has 'Names1' and 'Names2' columns

names1 = df['Names1']

names2 = df['Names2']

# Initialize an empty list to store the similarity scores

similarity_scores1 = []

similarity_scores2 = []

# Calculate the similarity scores for each name in 'Names1' with all names in 'Names2'

for name1 in names1:

scores1 = [fuzz.ratio(name1, name2) for name2 in names2]

similarity_scores1.append(scores1)

scores2 = [fuzz.partial_ratio(name1, name2) for name2 in names2]

similarity_scores2.append(scores2)

# Create a DataFrame with the similarity scores

similarity_df1 = pd.DataFrame(similarity_scores1, columns=names2)

similarity_df2 = pd.DataFrame(similarity_scores2, columns=names2)

# Set the index of the DataFrame to match the names in 'Names1'

similarity_df1.index = names1

similarity_df2.index = names1

# Save the DataFrame to an Excel file

similarity_df1.to_excel('similarity_matrix_1136_2959_LV.xlsx')

similarity_df2.to_excel('similarity_matrix_1136_2959_PR.xlsx')

print("Done")

**#Script - 4b**

import pandas as pd

# Read the similarity score matrix from your file

df = pd.read_excel('similarity_matrix_1136_2959_TSR.xlsx', index_col='Names1')

# Initialize an empty list to store dictionaries with names and scores between 80 and 100

selected_names_list = []

# Iterate through each row in the DataFrame

for index, row in df.iterrows():

# Extract names and scores greater than 80

high_scores_data = {name: score for name, score in row.items() if score >= 80}

# Remove names already present in 'High_Score_Data'

high_scores_data.pop(index, None)

# Add to the list

selected_names_list.append({'Names': index, 'High_Score_Data': high_scores_data})

# Create a DataFrame from the list of dictionaries

result_df = pd.DataFrame(selected_names_list)

# Save the DataFrame to an Excel file

result_df.to_excel('1136vs2959_greq80lessthan100_TSR.xlsx', index=False)

print("Selected names with scores between 80 and 100 have been saved to selected_names_output.xlsx")

**#Script - 4c**

import pandas as pd

import ast

# Read the Excel file

df = pd.read_excel('991_greq80lessthan100_LV_rough.xlsx', header=None, names=['High_Score_Data'])

# Function to process each row in the 'Original' column

def process_row(row):

try:

# Safely evaluate literal or expression from the cell value

data = ast.literal_eval(str(row['High_Score_Data']))

# Extract names from the dictionary

names = ', '.join(data.keys())

return names

except Exception as e:

return f"Error: {e}"

# Apply the processing function to create a new 'Processed' column

df['Processed'] = df.apply(process_row, axis=1)

# Save the result to a new Excel file

df[['Processed']].to_excel('output_file_LV.xlsx', index=False)

print("Done")

**#Script - 4d**

import openpyxl

# Specify the input Excel file name

input_file_name = 'input_122_1136vs2959.xlsx'

# Specify the output Excel file name

output_file_name = 'output_122_1136vs2959.xlsx'

# Open the Excel file and read the data

workbook = openpyxl.load_workbook(input_file_name)

sheet = workbook.active

# Extract data from the Excel sheet

data = [name.strip() for row in sheet.iter_rows(min_row=1, values_only=True) for name in row]

output_data = [name for names in data for name in names.split(',')]

# Create a new workbook for the output

output_workbook = openpyxl.Workbook()

output_sheet = output_workbook.active

# Write the output data to the new workbook

for index, name in enumerate(output_data, start=1):

output_sheet.cell(row=index, column=1, value=name)

# Save the output workbook

output_workbook.save(output_file_name)

print("Done")

#*****************************Script - 5********************

#The code reads a DataFrame from an Excel file containing trial data, iterates over continuous years, and creates a binary matrix indicating whether each #trial is active in a given year.

import pandas as pd

# Assuming your Excel file is named 'your_file.xlsx'

df = pd.read_excel('RB_108_MurugananthanK_StartDate_EndDate_3MonthsMargin.xlsx')

# Initialize an empty dictionary to store trial results

result_dict = {}

# Find the minimum and maximum years in the entire DataFrame

min_year = df['Start Date'].dt.year.min()

max_year = df['End Date'].dt.year.max()

# Create a continuous range of years

all_years = range(min_year, max_year + 1)

# Iterate over years in the continuous range

for year in all_years:

trial_results = {}

# Iterate over rows in the DataFrame

for index, row in df.iterrows():

# Check if the trial falls within the particular year

trial_results[row['Trial']] = int(row['Start Date'].year <= year <= row['End Date'].year)

# Add trial results to the dictionary

result_dict[year] = trial_results

# Convert the dictionary to a DataFrame

output_df = pd.DataFrame(result_dict)

# Write the result to an Excel file

output_df.to_excel('output_result_gantt_chart_continuous_years.xlsx')

print("Done")
